# Recombinant Lipoprotein Rv1016c Derived from *Mycobacterium tuberculosis* Is a TLR-2 Ligand that Induces Macrophages Apoptosis and Inhibits MHC II Antigen Processing

**DOI:** 10.3389/fcimb.2016.00147

**Published:** 2016-11-18

**Authors:** Haibo Su, Shenglin Zhu, Lin Zhu, Wei Huang, Honghai Wang, Zhi Zhang, Ying Xu

**Affiliations:** ^1^CAS Key Laboratory of Regenerative Biology, Joint of School of Life Science, Guangzhou Medical UniversityGuangzhou, China; ^2^Guangzhou Institutes of Biomedicine and Health, Chinese Academy of SciencesGuangzhou, China; ^3^State Key Laboratory of Genetic Engineering, Institute of Genetics, School of Life Science, Fudan UniversityShanghai, China; ^4^Department of Clinical Laboratory, Second People's Hospital of Guangdong ProvinceGuangzhou, China

**Keywords:** Rv1061c, MHC II, *Mycobacterium tuberculosis*, TLR2, apoptosis

## Abstract

TLR2-dependent cellular signaling in *Mycobacterium tuberculosis*-infected macrophages causes apoptosis and inhibits class II major histocompatibility complex (MHC-II) molecules antigen processing, leading to evasion of surveillance. *Mycobacterium tuberculosis* (MTB) lipoproteins are an important class of Toll-like receptor (TLR) ligand, and identified as specific components that mediate these effects. In this study, we identified and characterized MTB lipoprotein Rv1016c (lpqT) as a cell wall associated-protein that was exposed on the cell surface and enhanced the survival of recombinants M. smegmatis_Rv1016c under stress conditions. We found that Rv1016c lipoprotein was a novel TLR2 ligand and able to induce macrophage apoptosis in a both dose- and time-dependent manner. Additionally, apoptosis induced by Rv1016c was reserved in THP-1 cells blocked with anti-TLR-2 Abs or in TLR2^−/−^ mouse macrophages, indicating that Rv1016c-induced apoptosis is dependent on TLR2. Moreover, we demonstrated that Rv1016c lipoprotein inhibited IFN-γ-induced MHC-II expression and processing of soluble antigens in a TLR2 dependent manner. Class II transactivator (CIITA) regulates MHC II expression. In this context, Rv1016c lipoprotein diminished IFN-γ-induced expression of CIITA IV through TLR2 and MAPK Signaling. TLR2-dependent apoptosis and inhibition of MHC-II Ag processing induced by Rv1016c during mycobacteria infection may promote the release of residual bacilli from apoptotic cells and decrease recognition by CD4^+^ T cells. These mechanisms may allow intracellular MTB to evade immune surveillance and maintain chronic infection.

## Introduction

*Mycobacterium tuberculosis* (MTB) is considered to be the significant cause of mortality worldwide, especially due to its ability to resistant antibiotic and co-infected with HIV patients (Bruchfeld et al., 2015; Dheda et al., 2016). Host resistance against MTB infection depends on both innate and adaptive immunity, and the emerging evidences in mammals have indicated that CD4^+^T cells initiates the adaptive response and are critical to control of MTB infection, although CD8^+^T cells are also involved (Torrado and Cooper, 2011; Jasenosky et al., 2015). However, MTB remains to survival within infected macrophages for prolonged periods by evading the elimination of host immune responses (Holvast et al., 2010; Dorhoi and Kaufmann, 2014). The molecular basis that allow MTB to persist for years in the face of vigorous CD4^+^T cell responses may involve the decreased antigen processing or MHC-II expression in infected macrophages, which prevented the recognition of infected macrophages by effector CD4^+^T cells (Noss et al., 2000; Kaufmann et al., 2016). Actually, MTB indeed can inhibit antigen processing by murine macrophages via a mechanism involving decreased synthesis of MHC-II molecules, consistent with other reports of the ability of mycobacteria to decrease MHC-II expression by infected cells (Kaufmann and Schaible, 2005; Pecora et al., 2009; Satchidanandam et al., 2014).

*Mycobacterium tuberculosis* was capable of inducing macrophages to undergo apoptosis *in vitro*. Recently, emerging interest has arisen in the role of macrophage apoptosis in host defense against MTB infection (Keane et al., 2000). However, A variety of questions including the characterization of the responsible microbial factors and the underlying apoptotic mechanism involved remain unresolved. Knowledge about the mechanisms involved in the apoptosis of mycobacteria-infected macrophages suggested that *M. tuberculosis* could interact with macrophages and induced apoptosis through TLR signaling (Means et al., 2001). TLR-2 has been demonstrated to recognize *M. tuberculosis* or its subcellular fractions and activate intracellular apoptotic signaling. A few of molecularly diverse mycobacterial products involved in macrophage apoptosis have been identified; among these are 19-kDa lipoprotein (Lopez et al., 2003), ESAT6 (Derrick and Morris, 2007), lipomannan (Dao et al., 2004), and 38-kDa lipoprotein (Sanchez et al., 2009). Identification of *M. tuberculosis* factors responsible for inducing apoptosis will facilitate the understanding of pathogenesis and may provide novel strategies for MTB prevention or treatment.

TLR2 on APCs are critical for host protection and immunopathology, particular in host APCs sensing mycobacteria (Kleinnijenhuis et al., 2011). Previous studies showed that deficiency in TLR2 could enhance the susceptibility of mice to mycobacterial infection (Reiling et al., 2002; Gomes et al., 2004). The TLR2 polymorphisms in human are also associated with increased susceptibility to tuberculosis (Ogus et al., 2004; Texereau et al., 2005; Dalgic et al., 2011). So far, several mycobacterial ligands including lipoproteins have been found to bind to TLR2 (Pennini et al., 2006; Shi et al., 2009). MTB lipoproteins have either stimulatory or inhibitory effects on host antigen presenting cells (APCs), some of which are TLR2 dependent (Harding and Boom, 2010). Lipoprotein MPT83, a TLR2 ligand, promoted IFN-γ-induced MHC II expression and enhanced the ability of macrophages to present the MPT83 peptide to CD4^+^T by (Chen et al., 2012). However, prolonged exposure to other lipoproteins such as Lpr A, Lpr G as well as 19-kDa lipoproteins inhibited IFN-γ-induced MHC-II expression and antigen presentation via TLR2 (Pecora et al., 2006; Drage et al., 2010). Additionally, MTB lipoproteins inhibited several IFN-γ-induced immune function genes expression including II transactivator (CIITA), which regulates MHC II transcription, H2-M, and other accessory proteins required for Ag presentation, potentially inhibiting responses by CD4^+^T cells that produce IFN-γ (Noss et al., 2001). Consequently, a small group of infected macrophages with attenuated APC function may induce anti-inflammatory cytokines and be unable to present MTB antigens to CD4^+^T cells, providing a kind of mechanism by which MTB consistently persists and evades immune surveillance (Richardson et al., 2015).

Regulation of MHC-II expression or antigen processing can be achieved by exposure of macrophages to MTB components, which initiates responses by APCs that regulate both innate and adaptive immunity (De Lerma Barbaro et al., 1999). So far, only several MTB lipoproteins have been characterized to modulate this process. Here, we characterized a novel MTB lipoprotein Rv1016c, and determine its effects on host macrophages. Our results showed that Rv1016c prolonged the survival of recombinant *M. smegmatis* within host macrophages and accelerated host cell apoptosis. We present evidences that Rv1016c inhibited IFN-γ–induced antigen presentation and MHC-II expression in macrophages dependent on TLR2. We further demonstrated that Rv1016c lipoprotein inhibited IFN-γ-induced expression of CIITA IV via TLR2 and MAPK Signaling. Together, Rv1016c may involve mycobacterial virulence via a mechanism, which induced apoptosis and diminished antigen presentation to CD4^+^ T cells, contributing to immune evasion by *Mycobacterium tuberculosis*.

## Materials and methods

### Mice and cell lines

C57BL/6, TLR2 knockout, and TLR4 knockout mice were obtained from the Model Animal Research of Nanjing University (Nanjing, China), and they were housed under specific pathogen-free conditions in the Animal Center of the School of Life Science of Fudan University. All experimental procedures followed the Guidelines for the Care and Use of Laboratory Animals from the National Institutes of Health and were approved by the Animal Care and Use Ethical Committee of Shanghai Provence. THP-1 cell line was purchased from the Cell Bank of the Chinese Academy of Sciences (Shanghai, China). Cells were cultured in Dulbecco's modified Eagle's medium (DMEM) (Gibco, Grand Island, NY, USA) supplemented with 10% fetal bovine serum (FBS), penicillin (100 U/ml) and streptomycin (100 mg/ml) and maintained at 37°C in a humidified incubator (5% CO_2_). PBMCs were isolated from whole blood with Ficoll-Paque Plus (Amersham Biosciences, Piscataway, NJ). Monocytes were prepared from PBMCs either by adherence or with the Miltenyi monocyte isolation kit (Miltenyi Biotec, Auburn, CA), and monocytes derived macrophages (MDMs) were obtained as previously described (Dao et al., 2004). The resultant macrophages were used during the following week. DOBW T hybridoma cells were used to detect OVA_323−339_ (Pai et al., 2003).

### Abs and reagents

Middlebrook 7H9, 7H10, and OADC were obtained from Difco (Detroit, MI). Annexin V-PE was from BD PharMingen (Mississauga, Ontario, Canada). OVA:I-Ab complexes were detected with DOBW T hybridoma cells (Pai et al., 2003). Anti-His was obtained from antibodies (Santa Cruz Biotechnology, SantaCruz, USA). Anti-Rv1016c, Anti-Rv2145c and Anti-Rv3425 mouse polyclonal antibody were from C57BL/6 mouse immunized by synthesized respective peptides in our lab. The primary Abs used included rabbit antiERK2, rabbit anti-p38, rabbit anti-JNK, rabbit anti-phosphoERK1/2, rabbit anti-phospho-p38, rabbit anti-phospho-JNK, rabbit anti-phospho-IκBα, and peroxidase (HRP)-conjugated secondary Abs (Cell Signaling Technology). Anti-TLR2 and anti-TLR4 were from BioLegend. Alexa488-conjugated anti- His Abs, Alexa488-conjugated or Alexa555-conjugated secondary antibodies were obtained from (Cell Signaling Technology).

### Expression of recombinant Rv1016c and construction of *M. smegmatis*_Rv1016c

The MTB gene was amplified by PCR from H37Rv genomic DNA using the forward primer 5′CCCAAGCTTGGGGAGGGCATCCGGCGGGCTTG3′ and the reverse primer 5′CGGGATCCCGGCCCCAACCGTGCGGACAAT3′, which contain a Hind III site and a BamH I site, respectively. The amplified PCR product was cloned into the vector pRSFDuet-1 (Novagene, Madison, WI). The expression of the recombinant protein was induced by adding isopropyl-β-thiogalactopyranoside (IPTG) to a final concentration of 0.1 mM as previously described (Su et al., 2015). Recombinant Rv1016c was purified using a HIS-Select® Nickel Affinity Gel (Sigma-Aldrich, St. Louis, MO, USA) with minor modifications. Recombinant protein was dialyzed against PBS (pH 7.4) and treated with Pierce High Capacity Endotoxin Removal Resin (Pierce, USA) in accordance with the user instructions to eliminate endotoxins. The endotoxin-free Rv1016c protein was quantified with a bicinchoninic acid (BCA) protein assay kit (Pierce, Rockford, IL, USA) and frozen at −80°C. Samples were subjected to SDS-PAGE, and the His-tagged Rv1016c protein was detected by Western blotting using mouse anti-his antibody or anti- Rv1016c.

The pNIT plasmid containing Rv1016c was electroporated into *M. smegmatis*. The selected *M. smegmatis*_Rv1016c (Ms_Rv1016c) transformants were cultured in Middlebrook 7H9 with 10% oleic albumin dextrose catalase (OADC) containing 50 μg/ml kanamycin. The Ms_Rv1016c strain was identified by immunoblotting using anti- Rv1016c mouse polyclonal anti-serum. The vector pNIT was electroporated into *M. smegmatis* to generate Ms_ pNIT strain as a control.

### Localization of the Rv1016c protein

MTB H37Rv and Ms_Rv1016c were grown, and subcellular fractionation of Rv1016c protein was performed as previously described (Xu et al., 2015). Briefly, the cells were lysed by sonification for 30 min, and the lysates were centrifuged at 11,000 g for 5 min at 4°C to precipitate cellular debris and those unlysed cells. The supernatant was subjected to ultracentrifugation at 27,000 × g for 40 min at 4°C to recover cell wall-associated proteins. The supernatant was dell membrane and cytosol fractions. Each fraction was subjected to Western blotting using anti-Rv1016c mouse polyclonal antibody.

Proteinase K degradation assay was performed as previously described (Xu et al., 2015). Briefly, the recombinant Ms_Rv1016c were treated with proteinase K at a concentration of 100 μg/ml and incubated at 37°C for the indicated times. The reaction was stopped by the addition of protease inhibitor Cocktail Tablets (Roche, Basel, Switzerland). Finally, each sample was analyzed by Western blotting using anti-Rv1016c and anti-Rv2145c Abs (as a cytoplasmic protein control).

### Intracellular survival assay

THP-1 cells (1 × 10^6^ cells per well) were cultured in 6-well tissue plates. Following 48 h of treatment with 0.1 mg/ml of phorbol 12-myristate-13-acetate (PMA) (Sigma), THP-1 cells were transformed into an adherent state. Cells were infected with Ms_Rv1016c or Ms_pNIT at an MOI of 10. Four hours after infection, macrophages were incubated with a final concentration of 2 μg/ml hygromycin for 2 h. At indicated time after infection, macrophages were then washed twice and lysed in sterile water containing 0.025 % (v/v) SDS. The lysed cells were plated on 7H10 agar plates, and the colony forming units were determined as a measure of intracellular survival of recombinant *M. smegmatis*.

The survival of Ms_Rv1016c under different stress conditions was performed as previously described (Li et al., 2016). Briefly, the disk diffusion method was used to qualitatively measure the differences in H2O2 and SDS sensitivities between Ms_Rv1016c and Ms_pNIT. The indicated amount of H2O2 and SDS was spotted on 5.5 mm-diameter Whatman filter disks placed on the bacterial lawn. After 3–4 days incubation, the diameter of zone of complete inhibition was measured. All experiments were performed at least three times.

### Apoptosis assays

Apoptosis was assayed by either TUNEL assay or annexin V-PE staining as previously described (Lopez et al., 2003). TUNEL assays were analyzed using BD PharMingen kit (Mississauga, Ontario, Canada), according to the manufacturer's instructions. Cells were considered apoptotic if they were TUNEL positive (green fluorescent nuclear staining). Cells incubated with actinomycin D were as positive controls. For annexin V-PE assays, cells were treated with annexin V-PE buffer (10 mM HEPES, pH 7.4), and then incubated for 15 min at room temperature in the dark. After washed, and the volume of cells was increased to 400 μl with binding buffer for analysis by flow cytometry.

### TLR-binding assay

Macrophages isolated from wild type, TLR2^−/−^ and TLR4^−/−^ mouse were grown overnight on coverslips and then incubated for 30 min at 37°C with Rv1016c (20 μg/ml) in Hank's buffer. For flow cytometer, after washed with PBS and fixed for 15 min using 4% paraformaldehyde, the cells were stained with mouse anti-His IgG conjugated with Alexa 488 (Santa Cruz Biotechnology), then the cells were collected and 10,000 total events per sample were analyzed by using a BD FACScan calibrator (BD Biosciences, San Jose, CA). For confocal microscopy assay, macrophages were stained with anti-Rv1016c and anti-mouse TLR2 for 2 h at 37°C in PBS containing 2% BSA. After washing, the cells were stained with appropriate Alexa 488− or Alexa 555-conjugated secondary antibodies for 1 h at 37°C, and then they were incubated with 0.1 μg/ml DAPI for 5 min at room temperature. Finally, cells were mounted on slides using Mowiol solution (Sigma-Aldrich) and observed using a 63x oil objective on an LSM 710 microscope (Carl Zeiss, Munich, Germany).

### Immunoprecipitation assay

Macrophages from WT, TLR2^−/−^ or TLR4^−/−^ mouse were incubated with Rv1016c (20 μg/ml) for 6 h and lysed with RIPA lysis buffer (Sangon, China). The lysates were pre-cleared by adding protein A or G sepharose beads (Santa Cruz, CA, USA) for 2 h. After centrifugation at 10,000 × g for 5 min at 4°C, the supernatant was incubated with Isotype IgG, anti-TLR2 or anti-His overnight at 4°C. After harvested and washed, the beads were boiled in 5x sample buffer for 5 min. The proteins were separated on 10% SDS-PAGE and probed with anti-TLR2 (BioLegend, CA, USA), or anti-His Abs (Santa Cruz, CA, USA) as indicated, followed by incubation with HRP-conjugated mouse anti-rat or rabbit anti-mouse IgG Abs. Immunoreactive bands were detected using an ECL reagent (Thermo Fisher Scientific, MA, USA) and visualized by exposure to x-ray film.

### Detection of MHC-II expression by flow cytometry

MHC-II expression assay was performed as previously described (Noss et al., 2001). Macrophages (2 × 10^6^cells/well in a 6-well plate) were cultured for 24 h with 15 ng/ml IFN-γ, and then cultured for 24 or 48 h with IFN-γ with or without Rv1016c protein (20 μg/ml). Cells were harvested by vigorous pipetting and trypsinization, incubated for 30 min on ice with Fc Block (BD Pharmingen) at 1/100 in PBS with 0.1% BSA and for 1 h on ice with FITC-conjugated anti-MHC-II (e Bioscience), or isotype negative control Ig G (e Bioscience). After washed three times in PBS, cells were analyzed with a BD FACScan flow cytometer.

### Ag processing and presentation assays

Ag processing and presentation assays were performed as previously described (Noss et al., 2001). Macrophages (5 × 10^5^cells/well) were incubated with increasing concentrations of Rv1016c protein or control buffer in the presence of 15 ng/ml IFN-γ for 24 h. Then, cells were washed and incubated with indicated concentration of OVA_323−339_for 3 h. After fixed in 1% paraformaldehyde and washed extensively, the cells were finally incubated with DOBW T hybridoma cells (1 × 10^6^/well for 24 h). Supernatants from the T hybridoma assay were assessed for IL-2 using a CTLL-2 cell bioassay with a colorimetric determination using Alamar Blue (Alamar Biosciences, Sacramento, CA) and a Bio-Rad 550 microplate reader (Bio-Rad). Briefly, 5 × 10^3^ CTLL-2 cells in 50 μl of complete DMEM were incubated with 100 μl of culture supernatant for 16–20 h. Proliferation of CTLL-2 cells in each well was measured by a colorimetric assay using Alamar blue (15 μl; Trek Diagnostics, Westlake, OH) and a Versamax tunable microplate reader. Positive and negative controls were included in each experiment to monitor CTLL-2 cell responsiveness. All data presented for Ag processing assays compared controls with experimental conditions within the same experiment, measuring the effects of lipoprotein exposure relative to macrophages exposed to control buffer. IL-2 production was expressed as OD at 550 nm minus OD at 595 nm.

For IL-2 level assay, macrophages (1 × 10^5^cells/well) from wild type, TRL2^−/−^ or TRL4^−/−^ were incubated with Rv1016c (20 μg/ml) or medium in the presence of IFN-γ (15 ng/ml) for 24 h at 37°C. The cells were then washed with PBS and fixed for 20 min in in 1% paraformaldehyde. After Rv1016c antigen-specific CD4^+^ T cells were isolated from mice immunized with Rv1016c proteins, 1 × 10^6^cells were co-cultured with macrophages for 48 h at 37°C. Finally, the concentration of IL-2 in each supernatant sample was examined using ELISA according to manufacturer's instructions. All results of experiments represent the mean response of triplicate wells plus SD.

### Western blots

For Western blotting, equal amounts of protein were boiled in SDS-PAGE sample buffer under reducing conditions, and electrophoresed on 12% polyacrylamide gels. Protein was transferred to polyvinylidene difluoride membrane (Millipore), blocked in 5% BSA for 1 h at room temperature, and incubated with primary Abs in PBS with 0.1% Tween 20 overnight at 4°C, followed by incubation with horseradish peroxidase (HRP)-conjugated secondary Abs for 1 h at room temperature. The primary Abs included rabbit antiERK2, rabbit anti-p38, rabbit anti-JNK, rabbit anti-phosphoERK1/2, rabbit anti-phospho-p38, rabbit anti-phospho-JNK, and rabbit anti-phospho-IκB-α (Cell Signalling Technology). The secondary Ab was goat anti-rabbit IgG (Cell Signalling Technology). Pierce ECL Western Blotting Substrate was used to assay target bands (Thermo Fisher Scientific, Waltham, MA).

### Quantitative PCR assay of CIITA IV

Macrophages (2 × 10^6^cells/well) were incubated with or without Rv1016c (20 μg/ml), and then with IFN-γ (15 ng/ml) in the continued presence or absence of Rv1016c for various periods. Block of the MAPK signaling pathway experiments involved the pretreatment of THP-1 cells with inhibitors to p38 (SB203580, 10 μM), ERK (U0126, 10 μM), JNK (SP600125, 10 μM) for 1 h at 37°C, followed by incubation with Rv1016c for 36 h at 37°C. Total RNA was obtained using the RNeasy kit (Qiagen) and dissolved in RNase-free water. First-strand cDNA was synthesized using the SuperScript preamplification system (Life Technologies). The quantitative real-time PCR was performed using a high-speed thermal cycler (LightCycler; Roche Diagnostics, Indianapolis, IN), and product was assayed by FastStart Master SYBR Green I (Roche Molecular Biochemicals, Indianapolis, IN). All primers were synthesized by Sangon (Shanghai, China), and their sequences were as follows: CIITA IV sense, 5′-3′ACG CTT TCT GGC TGG ATT AGT; CIITA IV antisense, 5′-3′- TCA ACG CCA GTC TGA CGA AGG; GAPDH, sense, 5′–3′ AACGACCCCTTCATTGAC, and antisense, 5′–3′ TCCACGACATACTCAGCAC. RNA amount of genes were normalized to the level of GAPDH in each sample.

### Statistical analysis

All experiments were repeated 3 times at least with consistent results, which were calculated as the mean ± SD of 3 experiments. Statistical analysis was performed using a one-way ANOVA followed by Tukey's test using the origin 8.0 software (Origin Lab, USA). For all tests, *p* ≤ 0.05 was considered statistically significant.

## Results

### Rv1016c is associated with Mycobacterial cell wall

**Figure 1:**
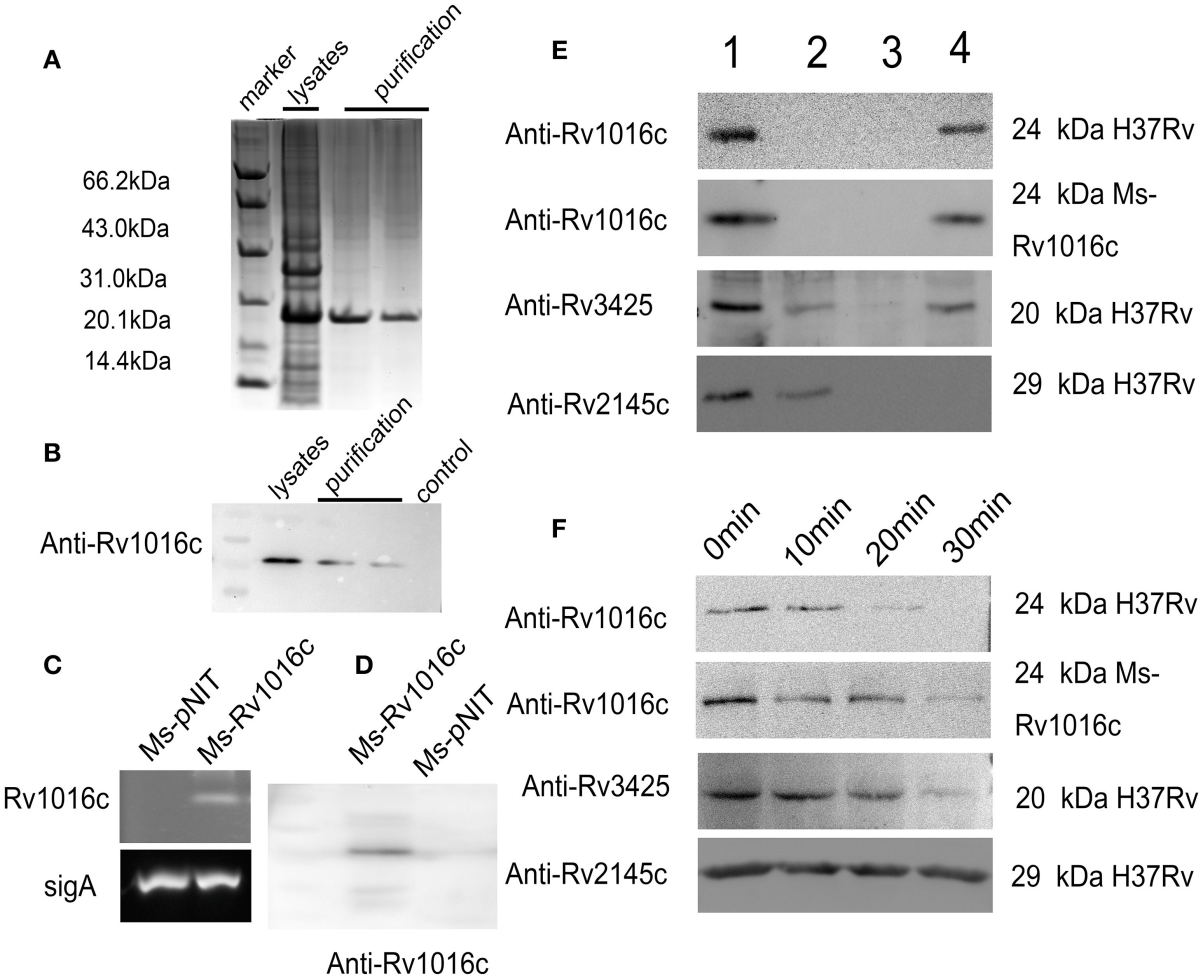
**Rv1016c is a cell Mycobacterial well-associated protein and exposes on cell surface. (A)** The expression of Rv1016c-His tag in *E.coli* BL21 strain was detected by commassie blue staining. **(B)** Western analysis of solubilized and purified Rv1016c protein with anti-His Ab. **(C)** Detection of Rv1016c expression in recombinant *M. smegmatis* using RT-PCR. **(D)** Western blot analysis was used to examine Rv1016c expression in recombinant *M. smegmatis* with anti- Rv1016c antibody. **(E)** *M. tuberculosis* H37Rv and *M. smegmatis* strains expressing Rv1016c were subjected to fractionation experiments, and the Rv1016c protein in different fractions was detected by Western blotting using anti-Rv1016c antibody. The expression of cytosolic Rv2145c (cytosolic) and Rv3425 (cell wall-associated protein) were also detected by anti-Rv2145c antibody or anti-Rv3425 antibody as a negative or positive control. Lane1: whole-cell lysate; lane 2: cytoplasmic membrane fraction; lane 3: cytosolic fraction; lane 4: cell wall fraction. **(F)** *M. tuberculosis* H37Rv and Ms_Rv1016c strains expressing were digested by proteinase K for the indicated times (0, 10, 20, and 30 min). The samples were analyzed for the expression of Rv1016c by Western blotting using anti-Rv1016c antibody. Rv2145c and Rv3425 were analyzed by Western blotting as a control.

To define whether Rv1016c is essential for MTB pathogenesis, the Rv1016c gene was firstly amplified from MTB H37Rv genome DNA to generate *E.coli* BL21_PET28_ Rv1016c strains expressed a 6x his-tagged Rv1016c protein (Figure 1A). Western blotting also confirmed that the Rv1016c protein (~24 kDa Rv1169c-His protein) was successfully produced from *E.coli* BL21_PET28_ Rv1016c (Figure 1B). Secondly, the recombinant *M. smegmatis* strains were used to investigate the role of Rv1016c. The semi-RT-PCR demonstrated that Rv1016c could only be detected in Ms_Rv1016c (Figure 1C). Western Blot analysis using the anti-Rv1016c antibody further confirmed only Ms_Rv1016c strain expressed Rv1016c protein in the cell lysates, while absent in Ms_pNIT strain (Figure 1D).

Finally, MTB H37Rv strain and recombinant Ms_Rv1016c were subjected to cell fractionation experiments, followed by Western blot analysis with anti-Rv1016c antibody, to determine the location of Rv1016c. The results showed that Rv1016c lipoproteins were present in the cell wall fraction, indicating that Rv1016c may be associated with the cell wall similar to the localization of Rv3425, as Rv2145c (cytoplasmic proteins) was detected only in the cytoplasm (Figure 1E). Proteinase K assays further confirmed the cell surface association of Rv1016c, as the Rv2145c was not degraded at all (Figure 1F). Taken together, these results demonstrate that Rv1016c may be a cell wall-associated lipoprotein and exposed at the cell surface.

### Rv1016c enhances the survival of the recombinant *M. smegmatis* within macrophages and promotes the resistance to stress factors

**Figure 2:**
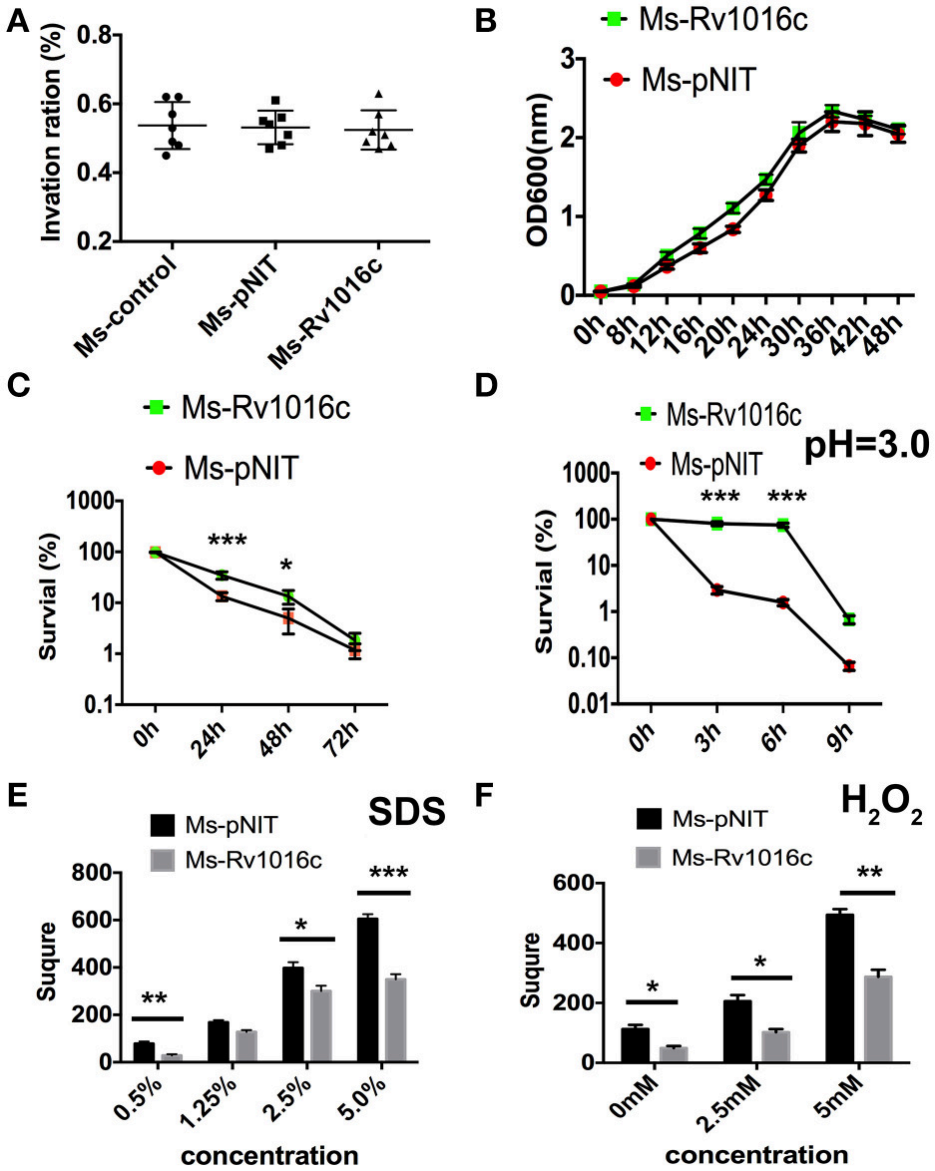
**Rv1016c promotes intracellular survival of ***M. smegmatis*** and boost the recombinants resistance to stress factors within macrophages. (A)** THP-1 cells were infected with Ms_pNIT or Ms_Rv1016c at an MOI of 10 at day 0 (4 h post-infection). Invasion ratio is the ratio of the number of intracellular bacteria and the number of initial infection bacteria. **(B)** Growth of Ms_Rv1195 and Ms_pNIT strains at 37°C in 7H9 liquid medium were assayed by OD600 value in every 4 h. **(C)** THP-1 cells were infected with Ms _pNIT or Ms_Rv1016c at the MOI of 10. After infection, Lysates containing the live bacteria were diluted gradually and then plated on 7H10 agar plates to determine bacteria number. **(D)** Growth of Ms _pNIT or Ms_Rv1016c under Low pH (pH = 3) was measured *in vitro*. The bacteria were collected by centrifugation and resuspended to an OD600 of 0.8 in 20 ml 7H9. The cultures were incubated at 37°C and 0.1 ml removed for viable count enumeration. **(E,F)** Growth of Ms _pNIT or Ms_Rv1016c under stress conditions. Disk diffusion method was used to measure the survival of recombinant Ms_Rv1016c when exposure to SDS **(E)** and H2O2 **(F)**. All results were generated from three independent experiments and Data are shown as Means ± SD of triplicate wells. (^*^p < 0.05, ^**^p < 0.01, ^***^p < 0.001, determined by Student's *t*-test).

To examine whether Rv1016c can modulate the intracellular mycobacterial invasion, growth and survival, we compared the growth rate of Ms_Rv1016c and Ms_pNIT in macrophages. The infection experiment was carried out at an MOI of 10 (10 bacterium per 1 macrophages). Our results demonstrated that macrophages infected with Ms_pNIT and Ms_Rv1016c show the same invasion ratio (Figure 2A), and there was no significant difference in growth rates of the two strains (Figure 2B). However, Ms_Rv1016c showed significantly higher bacillary counts in THP-1 cells 24–72 h after infection (Figure 2C). The results demonstrate that the presence of Rv1016c does not influence uptake but can enhance the intracellular survival of *M. smegmatis* within macrophages.

To further determine how Rv1195 enhanced the intracellular survival in macrophages, we compared the survival rate of the Ms_pNIT and Ms_Rv1016c under stresses *in vitro*. As shown in Figure 2D, the survival percentage of Ms_Rv1016c was significantly higher than Ms_pNIT under acid stress (pH = 3). For reactive oxygen resistance assay, the square of the zone was smaller in Ms_Rv1016c than those of the Ms_pNIT after H_2_O_2_ treatment (Figure 2E). Moreover, the percentage of survival of Ms_Rv1016c was significantly higher than that of Ms_pNIT after SDS treatment (Figure 2F). These findings indicate that Rv1016c may be involved in virulence, leading to increased resistance to stresses during mycobacteria infection.

### Rv1016c is a ligand for TLR2

**Figure 3:**
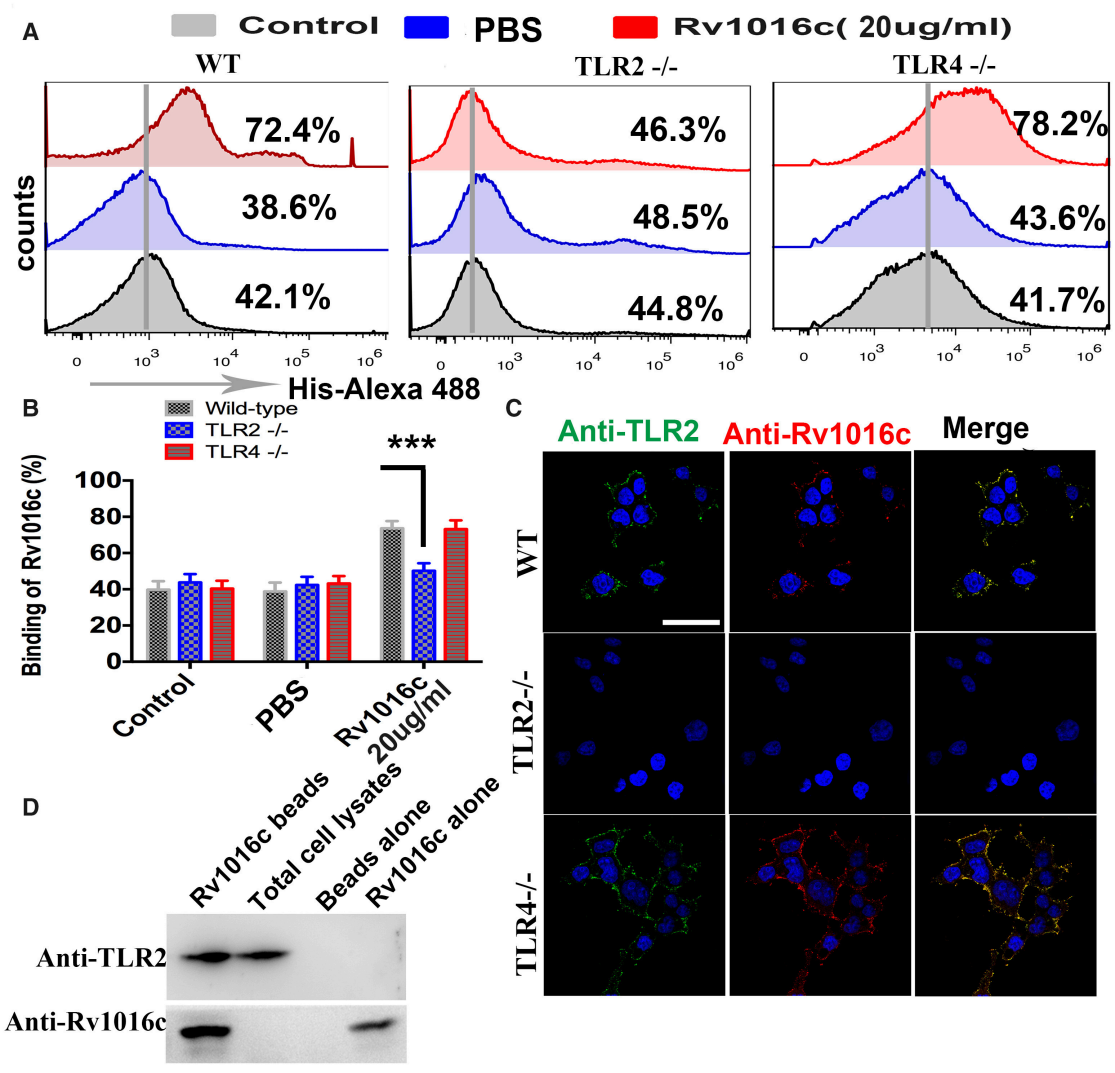
**Rv1016c interacts with TLR2, but not with TLR4. (A,B)** Macrophages derived from WT, TLR2^−/−^, and TLR4^−/−^ mice were incubated with Rv1016c-His (20 μg/ml) for 1 h, followed by incubation with Alexa488-conjugated anti-His Abs. After washing and staining, flow cytometry was used to examine the binding between Rv1016c and TLR2. **(C)** Macrophages derived from WT, TLR2^−/−^, and TLR4^−/−^ mice were treated with Rv1016c (20 μg/ml) for 1 h, fixed and stained with primary antibodies (mouse anti-Rv1016c, rat anti-mouse TLR2), followed by incubation with appropriate Alexa488- or Alexa555-conjugated secondary antibodies. Cells were photographed under fluorescence microscopy (original magnification × 63). Scale bar = 10 μm **(D)** Cells were incubated with Rv1016c immobilized on Ni-NTA beads for 6 h, and proteins were visualized by Western blotting with anti-His or anti-TLR2 Abs to assay the presence of TLR2 with bead bound proteins. All data were generated from three independent experiments and Data are shown as Means±SD of triplicate wells. (^***^p < 0.001, determined by Student's *t*-test).

Although recognition of mycobacterial lipoproteins such as Lpq H, Lpr G, and Lpr A are known to signal through TLR, Rv1016c has not been tested for TLR ligand activity. To analyze whether Rv1016c can bind to TLR2, macrophages from WT, TLR2^−/−^ and TLR4^−/−^ mice were incubated for 24 h with Rv1016c. Cells were then stained with mouse anti-His IgG conjugated with Alexa 488. Flow cytometry analysis showed that Rv1016c bound to the surface of WT and TLR4^−/−^ mouse macrophages but not to the surface of TLR2^−/−^ cells (Figures 3A,B). Immunofluorescence analysis also showed strong fluorescence of anti-Rv1016c on the surface of WT, and TLR4^−/−^ mice cells exposed to Rv1016c but no fluorescence on the surface of TLR2^−/−^ macrophages cells (Figure 3C). To further ascertain whether Rv1016c physically binds to the TLR2 molecule, we conducted an immunoprecipitation assay using whole-cell extracts from THP-1 cells. Western blots probed with anti-TLR2 showed that Rv1016c was able to interact with TLR2. No band was visible in the beads alone group or Rv1016c alone group (Figure 3D). These results demonstrate that Rv1016c interacts specifically and predominantly with TLR2.

### Rv1016c induces macrophages apoptosis through TLR2

**Figure 4:**
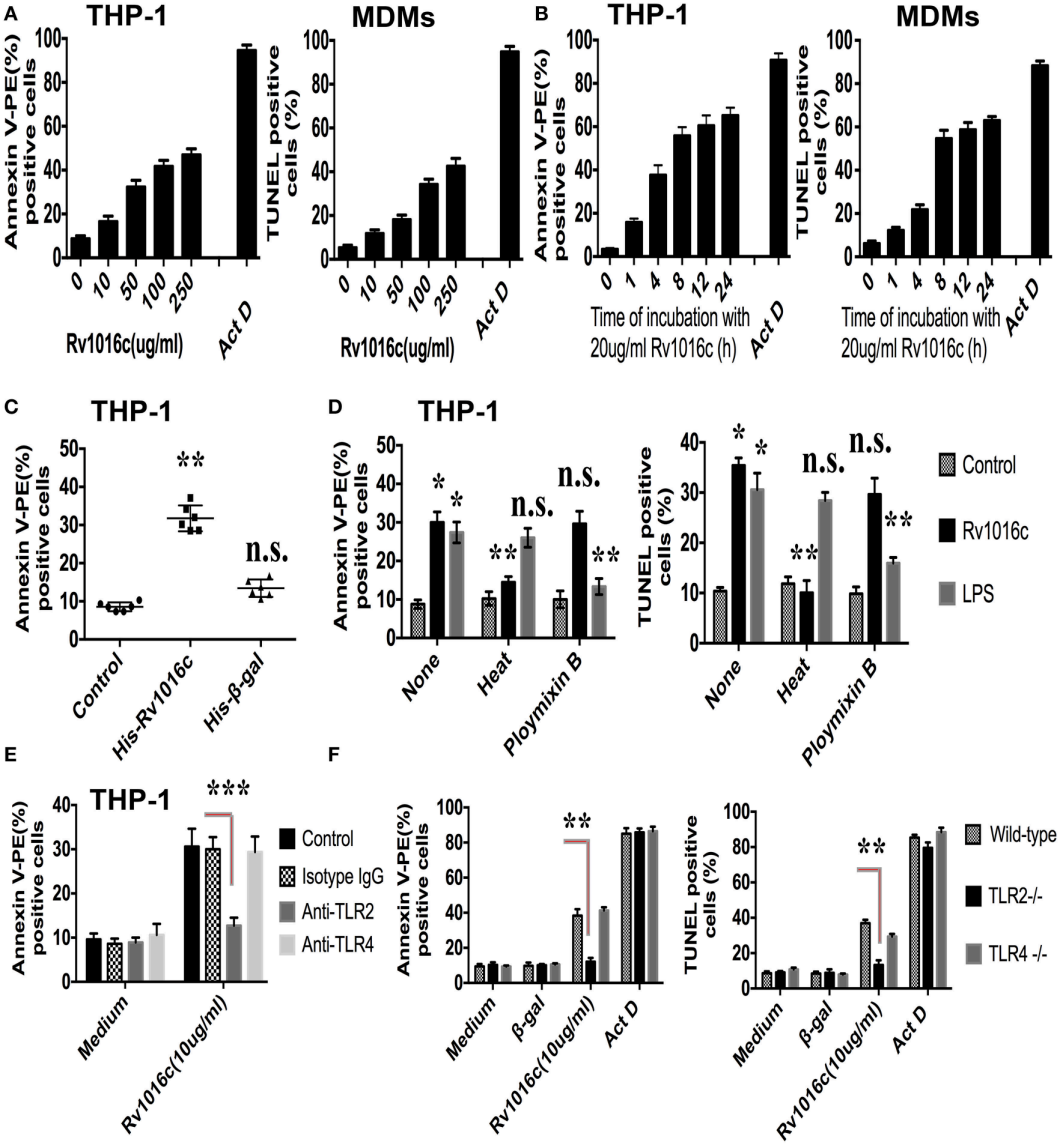
**Rv1016c induces apoptosis through TLR2 in macrophages**. THP-1 cells or monocytes derived macrophages (MDMs) were incubated with either a range of concentrations of Rv1016c for 2 h **(A)** or for varying lengths of time with 20 μg/ml recombinant protein **(B)**. Apoptosis was analyzed by flow cytometry or TUNEL assay. Actinomycin D was used as a positive control for apoptosis. **(C)** Apoptosis induced by Rv1016c is not due to His tag. THP-1 cells were incubated with equal amounts of His-tagged β-galactosidase (as control for specificity) or His-tagged Rv1016c. Apoptosis was analyzed by flow cytometry. **(D)** Apoptosis induced by Rv1016c is not due to LPS contamination. THP-1 cells were incubated with recombinant Rv1016c or LPS. Before addition to cells (20 μg/ml for 2 h), His-tagged Rv1016c was either subjected to boiling for 1 h or treated with polymyxin B resin. For the latter, 100 μl of polymyxin bead slurry was added to 100 μl of recombinant Rv1016c preparation and incubated at 4°C for 1 h. After centrifugation, the supernatant was used for apoptosis induction analyzed by flow cytometry or TUNEL assay. **(E)** THP-1 cells were incubated with Isotype Ig G (30 μg/ml), Anti-TLR2 blocking Abs (30 μg/ml) or Anti-TLR4 blocking Abs (30 μg/ml) for 18 h, followed by treated with 20 μg/ml recombinant Rv1016c for 2 h. Apoptosis was analyzed by flow cytometry. **(F)** Macrophages derived from C57BL/6, TLR2^−/−^, and TLR4^−/−^ mice were incubated with Rv1016c-His (20 μg/ml) for 2 h, and then apoptosis was analyzed flow cytometry or TUNEL assay. Incubation of cells with actinomycin D or Histagged β-galactosidase was used as a control for apoptosis. All data were generated from three independent experiments and Data are shown as Means ± SD of triplicate wells. (^*^p < 0.05, ^**^p < 0.01, ^***^p < 0.001 vs. corresponding controls).

To detect whether Rv1016c induces macrophages apoptosis, THP-1 cells or monocytes derived macrophages (MDMs) were treated with a range of concentrations of recombinant Rv1016c. Apoptosis was determined by flow cytometric analysis of annexin V-PE binding or by TUNEL assay. As shown in Figure 4A, increased levels of apoptosis were apparent with 20 μg/ml Rv1016c and continued to increase through 250 μg/ml, indicating that Rv1016c significantly induced apoptosis both in THP-1 cells and MDMs in a dose dependent manner. Apoptosis kinetics analysis showed that 20 μg/ml Rv1016c significantly induced apoptosis as early as 1 h after exposure and continued to increase through 24 h (Figure 4B). Recombinant Rv1016c–induced apoptosis was specific, as the frequency of annexin V-PE-positive cells after treatment with the Histagged β-galactosidase was similar to that observed for untreated control cells (Figure 4C). In addition, to exclude whether LPS contamination leads to apoptosis, the recombinant Rv1016c protein was exposed to either heat treatment or to polymyxin B before addition to cells. As shown in Figure 4D, LPS- induced apoptosis changed little due to its resistant to heat treatment not its sensitive to polymyxin B. In contrast, the apoptotic effects of recombinant Rv1016c protein were completely abrogated after heat treatment. We further determined the role of TLR2 in Rv1016c-induced apoptosis. THP-1 cells were incubated with Isotype IgG, anti-TLR2 Abs or anti-TLR4 Abs for 18 h before Rv1016c treatment, and apoptosis was analyzed as previously described. As shown in Figure 4E, incubation with anti-TLR2 Abs significantly abrogated the proapoptotic effects of Rv1016c lipoprotein. Similarly, when compared with wild type or TLR4 knockout cells, apoptosis induced by Rv1016c was completely abrogated in TLR2 knockout cells (Figure 4F). Taken together, these results indicate that Rv1016c-induced apoptosis is dependent on TLR-2.

### Rv1016c is able to inhibit IFN-γ-induced expression of MHC-II in macrophages

**Figure 5:**
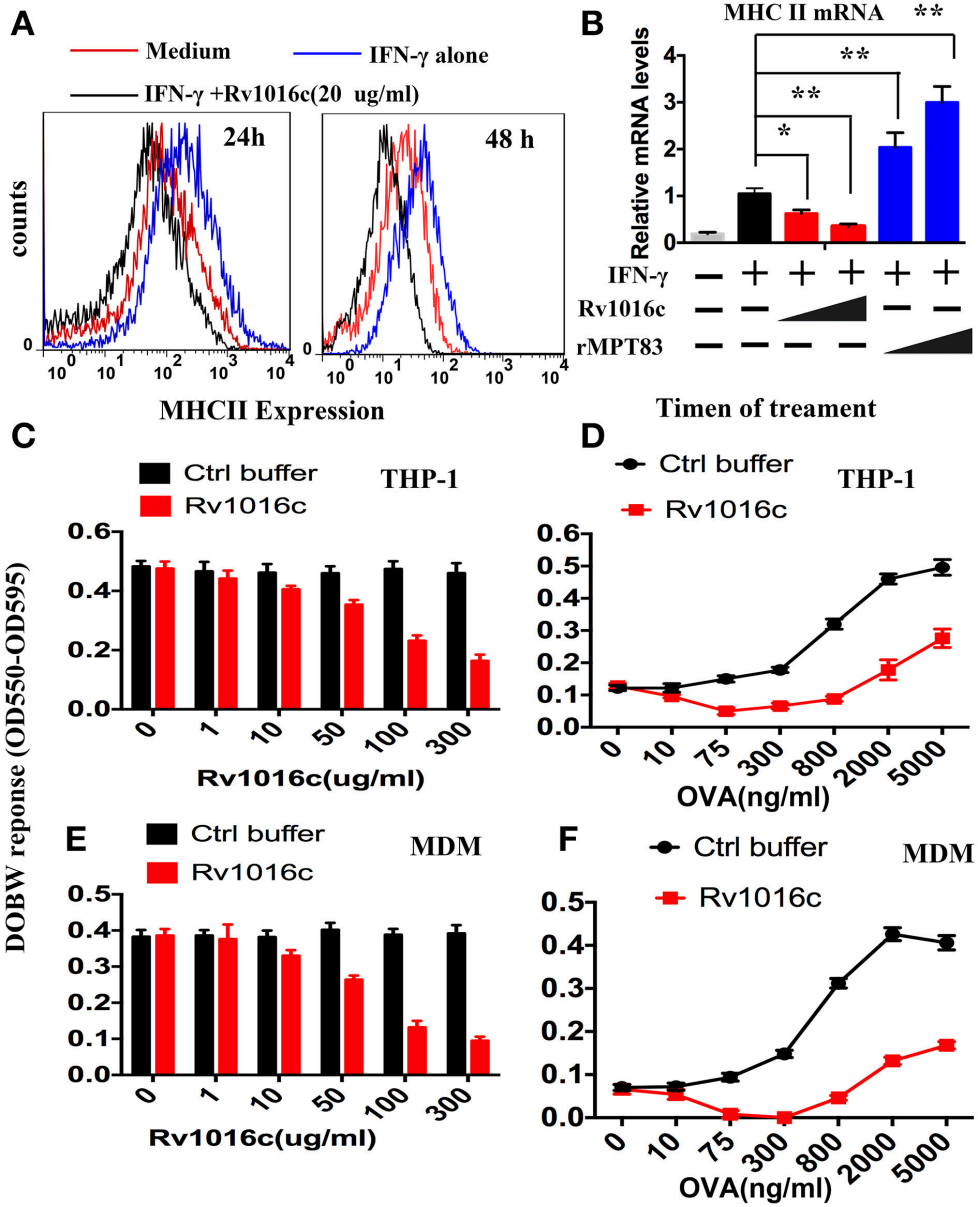
**Rv1016c inhibits IFN-γ-induced MHC-II expression and Ag processing. (A)** THP-1 cells were stimulated with IFN-γ (15 ng/ml) for 24 h at 37°C, and then treated with or without Rv1016c-His (20 μg/ml) for 24 (left) or 48 h (right). Cells were incubated with FITC-conjugated anti-MHC II, or isotype Ig G control Abs for 30 min in the dark room. MHC-II expression was analyzed with a BD FACScan flow cytometer. **(B)** THP-1 cells were incubated with medium, Rv1016c (20 μg/ml, 50 μg/ml) or rMPT8 (20 μg/ml, 50 μg/ml, as a positive control) in the presence of with IFN-γ (15 ng/ml). MHC II mRNA levels were measured by qPCR. **(C)** THP-1 (1 × 10^6^ cells /well) were incubated with increasing concentrations of Rv1016c or control buffer for 24 h in the presence of IFN-γ (15 ng/ml). Then, cells were washed and incubated with OVA_323−339_ (800 ng/ml) for 3 h. After fixed in 1% paraformaldehyde and washed extensively, the cells were finally incubated with DOBW T hybridoma cells (1 × 10^6^/well for 24 h). Supernatants from the T hybridoma assay were assessed for IL-2 using a CTLL-2 cell bioassay. **(D)** THP-1 cells were incubated with 100 μg/ml Rv1016c in the presence of IFN-γ (15 ng/ml), After 24 h incubation, cells were pulsed with increasing concentrations of OVA_323−339_ for 3 h. Ag processing was measured with DOBW T hybridoma cells as previously described. **(E)** HLA-DR1-positive MDMs were treated with increasing concentrations of Rv1016c or control buffer for 24 h. Following incubation with Rv1016c, macrophages were pulsed with OVA_323−339_ (800 ng/ml) for 3 h. Ag processing was measured with DOBW T hybridoma cells as previously described. **(F)** HLA-DR1-positive MDMs were incubated with 100 μg/ml Rv1016c in the presence of IFN-γ (15 ng/ml), After 24 h incubation cells were pulsed with indicated concentration of OVA_323−339_ for 3 h and fixed. Ag processing was measured with DOBW T hybridoma cells as previously described. Data points represent the mean of triplicate samples with SD and each panel is representative of at least two independent experiments. (^*^p < 0.05, ^**^p < 0.01 vs. corresponding controls).

Prolonged incubation of macrophages with mycobacterial lipoproteins modulates IFN-γ-induced MHC-II expression and antigen-presentation activity (Pennini et al., 2006). To clarify whether the Rv1016c lipoprotein regulates MHC-II expression, IFN-γ-stimulated THP-1 cells were treated with or without Rv1016c for 24–48 h, and then flow cytometry was used to analyze MHC-II expression. As shown in Figure 5A, the level of MHC-II was low in Rv1016c-pusled macrophages compared to cells cultured in medium, indicating that Rv1016c lipoprotein inhibited IFN-γ–enhanced MHC-II expression. Similar observations were also made for the endogenous MHC II mRNA levels in THP-1 cells (Figure 5B). To further assess whether Rv1016c affects the ability of Ag processing and presentation of mouse macrophages, THP-1 cells or monocyte-derived macrophages (MDMs) were incubated for 24 h with or without Rv1016c, exposed to OVA_323−339_ for 3 h, fixed, and incubated with DOBW T hybridoma cells for 24 h. IL-2 secretion was determined using a CTLL-2 assay. The results showed that exposure of macrophages to increasing concentrations of Rv1016c for 24 h inhibited MHC-II Ag processing of OVA_323−339_(Figures 5C,E). Kinetic analysis demonstrated that substantial inhibition was first noted at 300 ng/ml with almost complete inhibition by 2000 ng/ml OVA_323−339_ (Figures 5D,F). These findings may identify Rv1016c as an inhibitor of MHC-II expression and Ag processing.

### Rv1016c inhibits MHC-II Ag processing dependent on TLR-2

**Figure 6:**
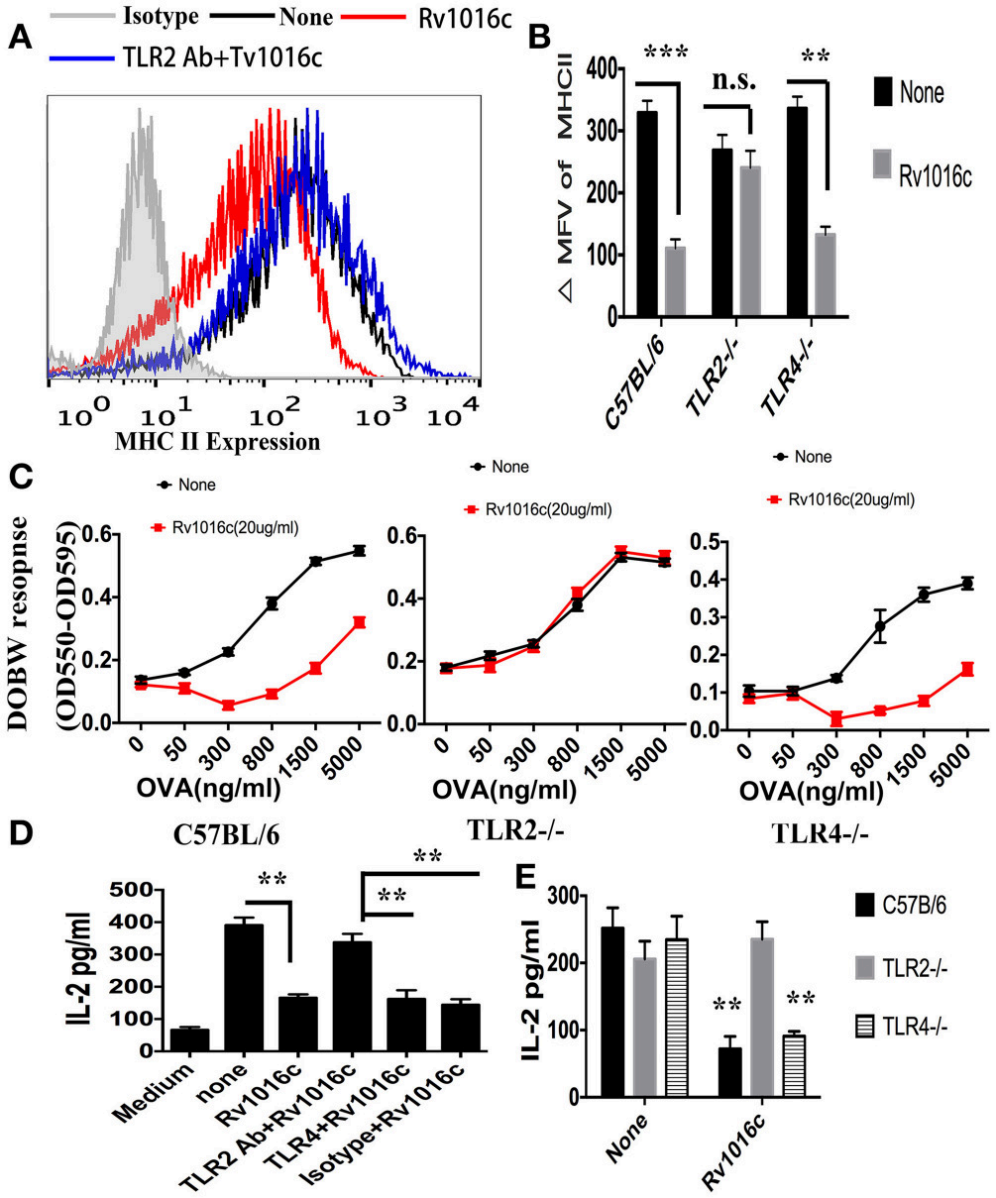
**Inhibition of MHC-II expression and Ag processing by Rv1016c lipoprotein is dependent on TLR 2. (A)** THP-1 were pretreated with Isotype Ig G (30 μg/ml) or Anti-TLR2 blocking Abs (30 μg/ml) for 18 h, followed by treated with IFN-γ (15 ng/ml) for 24 h at 37°C, and then treated with or without Rv1016c-His (20 μg/ml) for 24 h. Cells were then incubated with FITC-conjugated anti-MHC II or isotype Ig G control Abs for 30 min in the dark room. MHC-II expression was washed and analyzed with a BD FACScan flow cytometer. **(B)** Macrophages from WT, TLR2^−/−^ or TLR4^−/−^ mice were stimulated with IFN-γ (15 ng/ml) for 24 h at 37°C, and then treated with or without Rv1016c-His (20 μg/ml) for 24 h. MHC-II expression was measured as previously described. ΔMFV was calculated as the MFV of cells stained with anti–MHC-II minus the MFV of cells stained with the isotype-control Ab. **(C)** Macrophages from WT, TLR2^−/−^ or TLR4^−/−^ mice were stimulated with IFN-γ (15 ng/ml) for 24 h at 37°C, and then treated with or without Rv1016c-His (20 μg/ml) for 24 h. Then, cells were washed and incubated with indicated concentration of OVA_323−339_ for 6 h. After fixed in 1% paraformaldehyde and washed extensively, the cells were finally incubated with DOBW T hybridoma cells for 24 h. Supernatants from the T hybridoma assay were assessed for IL-2 using a CTLL-2 cell bioassay. **(D)** THP-1 cells (1 × 10^5^cells/well) were pretreated with Isotype Ig G (30 μg/ml), Anti-TLR2 blocking Abs (30 μg/ml) or Anti-TLR4 blocking Abs (30 μg/ml) for 18 h, and then incubated with or without Rv1016c (20 μg/ml) in the presence of IFN-γ (15 ng/ml) for 24 h at 37°C. After washed with PBS and fixed in 1% paraformaldehyde, Rv1016c Ag-specific CD4^+^ T cells (1 × 10^6^cells/well) were added to the plates, and were co-cultured for 48 h at 37°C. ELISA was used to assay the level of IL-2 in supernatant. **(E)** Macrophages (1 × 10^5^cells/well) from wild type, TRL2^−/−^ or TRL4^−/−^ mice were incubated with Rv1016c (20 μg/ml) or medium in the presence of IFN-γ (15 ng/ml) for 24 h at 37°C. Cells were washed with PBS and fixed for 20 min in 1% paraformaldehyde. Then, 1 × 10^6^cells/well Rv1016c antigen-specific CD4^+^ T cells were co-cultured with macrophages for 48 h at 37°C. IL-2 level was assayed as previously described. All data were generated from three independent experiments and Data are shown as Means ± SD of triplicate wells. (^**^p < 0.01, ^***^p < 0.001 vs. corresponding controls).

To examine whether Rv1016c inhibits MHC-II Ag processing through TLR2, THP-1 cells were pretreated with Isotype Ig G (30 μg/ml) or Anti-TLR2 blocking Abs (30 μg/ml) for 18 h, followed by treated with IFN-γ (15 ng/ml) for 24 h at 37°C, and then treated with or without Rv1016c-His (20 μg/ml) for 24 h, flow cytometry was finally used to analyze. As shown in Figure 6A, Rv1016c-mediated inhibition of IFN-γ-induced MHC-II expression was reversed in those macrophages, which were incubated with blocking anti-TLR-2 Abs. Additionally, Rv1016c lipoprotein decreased expression of MHC-II by C57BL/6 and TLR4^−/−^ mice but not TLR2^−/−^ mice (Figure 6B). To further assess whether Rv1016c affects the ability of Ag processing and presentation via TLR2, macrophages from wild type, TLR2^−/−^ or TLR4^−/−^ mouse were incubated for 24 h with or without Rv1016c (20 μg/ml), exposed to a range concentration of OVA_323−339_ for 3 h, fixed, and incubated with DOBW T hybridoma cells. IL-2 secretion was determined using a CTLL-2 assay. Inhibition of MHC-II Ag processing of OVA_323−339_ was reserved in TLR2^−/−^ mouse macrophages (Figure 6C). Furthermore, THP-1 cells were pretreated with Isotype Ig G, Anti-TLR2 blocking Abs or Anti-TLR4 blocking Abs for 18 h, followed by treated with or without Rv1016c (20 μg/ml) for 24 h in the presence of IFN-γ (15 ng/ml), and then incubated with Rv1016c-specific splenic CD4^+^ T cells. Supernatant levels of IL-2 were measured using ELISA. The result showed that Rv1016c-induced decrease in MHC-II presentation of Rv1016c peptide was blocked by anti-TLR2 Abs but not by anti-TLR4 or the isotype Abs, as reflected by increased IL-2 levels (Figure 6D). Similarly, Rv1016c inhibited the processing and presentation of Rv1016c peptide by C57BL/6 and TLR4^−/−^macrophages but not TLR 2^−/−^ macrophages (Figure 6D). These findings indicate that Rv1016c lipoprotein may inhibit expression of MHC-II molecules and the processing of Ag for presentation to CD4^+^ cells dependent on TLR2, independent on TLR4.

### Rv1016c inhibits IFN-γ-induced CIITA expression by TLR2 and MAPK signaling

**Figure 7:**
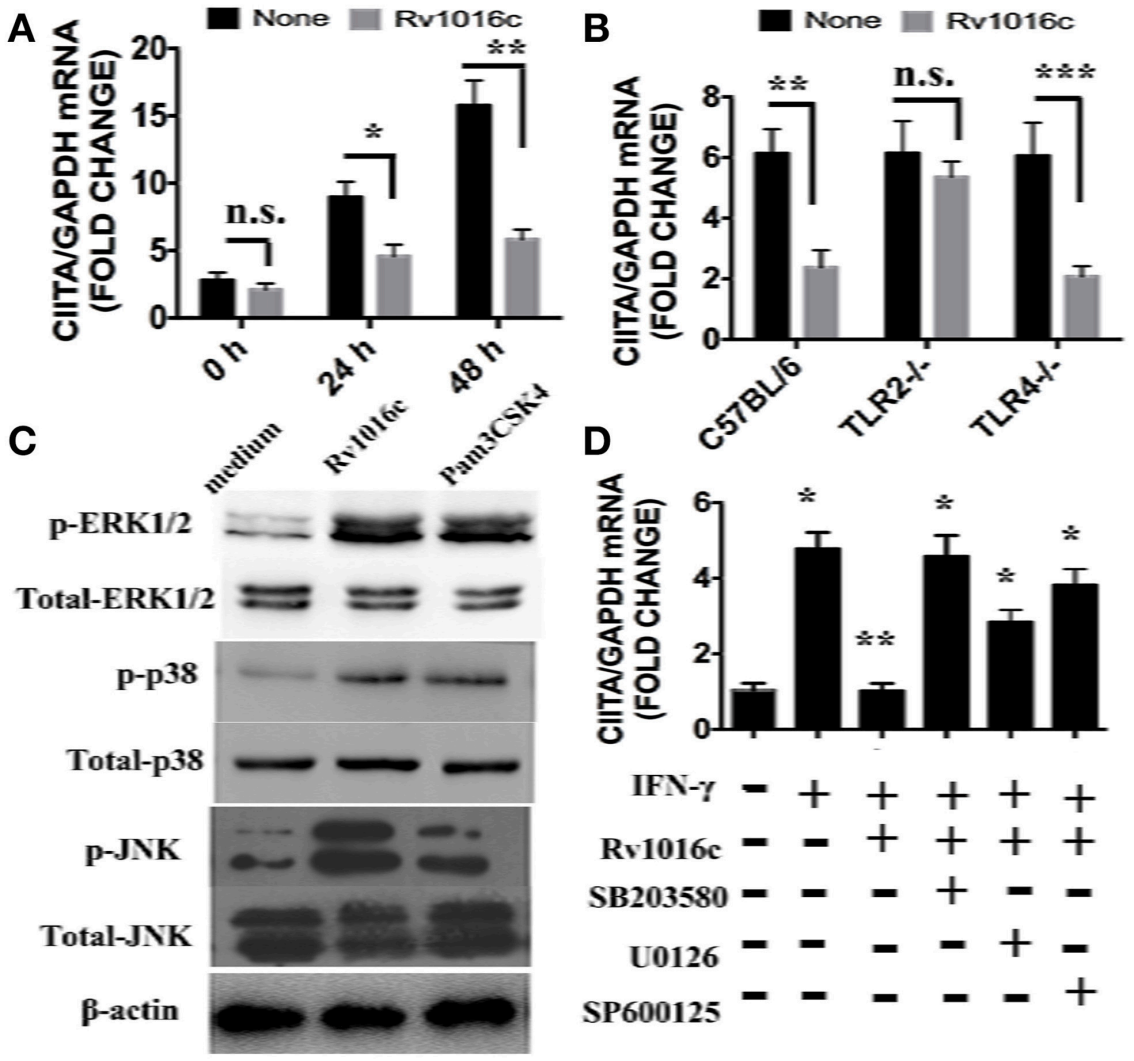
**Rv1016c inhibits IFN-γ- induced expression of CIITA dependent on TLR2 and MAPK pathway. (A)** THP-1 cells were incubated with or without Rv1016c-His (20 μg/ml) for 24 h, then with IFN-γ (15 ng/ml) in the continued presence or absence of Rv1016c for the indicated times. RNA was isolated, and quantitative real-time PCR was used to analyze the expression of CIITA. **(B)** Macrophages (1 × 10^6^cells/well) from wild type, TRL2^−/−^ or TRL4^−/−^ were incubated with or without Rv1016c-His (20 μg/ml) for 24 h, then with IFN-γ (15 ng/ml) in the continued presence or absence of Rv1016c-His for the indicated times. The expression of CIITA was measured as previously described. **(C)** Rv1016c activates MAPKs pathway in macrophages. THP-1 cells were treated with Rv1016c (20 μg/ml) or Pam3CSK4 (5 μg/ml) for 1 h, the phosphorylation of p38, ERK (1/2) and JNK were examined by blotting with specific antibodies to p-p38, p38, p-ERK1/2, ERK1/2, p-JNK, and JNK. **(D)** Rv1016c-mediated inhibition of CIITA expression is dependent on MAPK signaling. Macrophages were incubated with or without DMSO (vehicle control), p38 (SB203580, 10 μM), ERK (U0126, 10 μM), or JNK (SP600125, 10 μM) for 1 h at 37°C. Rv1016c (20 μg/ml) was added to samples for the next 6 h, and IFN-γ (15 ng/ml) was added for a final 5 h (presence of inhibitors maintained). Expression of CIITA mRNA was assayed as previously described. All samples were first normalized to GAPDH and are expressed as the fold change compared with untreated controls. All data are expressed as the mean ± SD from three separate experiments. (^*^p < 0.05, ^**^p < 0.01, ^***^p < 0.001 vs. corresponding controls).

As an essential IFN-γ-associated immuneregulator, class II transactivator (CIITA) IV regulates MHC II expression. Previous study demonstrated that 19-kDa lipoprotein inhibited the expression of CIITA induced by IFN-γ. To determine whether Rv1016c has similar effects on CIITA IV, we incubated macrophages with Rv1016c for 18 h, and then treated the cells with IFN-γ for the indicated time. As shown in Figure 7A, CIITA expression induced by IFN-γ was inhibited by Rv1016c treatment. In addition, we used macrophages from C57BL/6, TLR2^−/−^ or TLR4^−/−^ mice to examine whether inhibition of CIITA IV requires TLR2 signaling through. Q-PCR results showed that the ability of Rv1016c to inhibit IFN-γ-induced CIITA expression was abrogated in TLR2^−/−^ macrophages (Figure 7B).

MAPK signaling is the key mediators of TLR2 pathway. In this context, MAPK was activated by Rv1016c incubation, as reflected by the increased phosphorylation of p38, ERK, and JNK (Figure 7C). To further determine whether MAPK plays a key role in Rv1016c-mediated inhibition of CIITA expression, we treated macrophages with p38 inhibitor (SB203580), ERK1/2 inhibitor (U0126), JNK inhibitor (SP600125) for 1 h prior to stimulation with Rv1016c. Levels of CIITA IV mRNA expression induced by IFN-γ was measured by Q-PCR. As shown in Figure 7D, three inhibitors could attenuate Rv1016c-mediated inhibition of CIITA IV expression. These results indicate that Rv1016c may inhibit CIITA IV expression dependent on TLR2 and MAPK signaling.

## Discussion

MTB genome encodes about 100 putative lipoproteins, which may have critical functions ranging from interfering mycobacterial virulence, generating antigenic variation and evading host immune responses (Sutcliffe and Harrington, 2004). LppX is involved in the correct localization of phthiocerol dimycocerosates (PDIMs), an important virulence factor of M. tuberculosis. LprG binds triacylated phospho-inositol-mannoside (PIM), lipomannan (LM) and lipoarabinomannan (LAM) involved in the inhibition of phagosome-lysosome fusion (Drage et al., 2010; Martinot et al., 2016). However, several mycobacterial lipoproteins form immunodominant antigens can promote protective immune response (Becker and Sander, 2016). Immunization with recombinant MPT83 or DNA-Mpt83 developed a strong IFN-γ secreting CD4+ T-cell response, and significantly decreased the bacterial load in lung and spleen as compared to control mice (Chen et al., 2012). The interaction between mycobacterial lipoproteins and host cells involved Toll-like receptor 2 (Pecora et al., 2006; Chambers et al., 2010), which plays an important role in the elaboration of protective Ag processing and inflammatory cytokines (Zeytun et al., 2007; Saraav et al., 2014). As the key roles of lipoproteins and TLR2 during MTB infection, the identification of novel MTB lipoproteins, which bind to TLR2, contributes to deep understanding the interaction between the pathogen and APCs where it resides (Drage et al., 2009). In this context, we identified Rv1016c as a mycobacterial cell wall -associated lipoprotein. In addition, the affinity column pull downs using Rv1016c immobilized on Ni-NTA beads followed by western blotting analysis with anti-TLR2 antibody confirmed that Rv1016c physically interacted with TLR2. Moreover, Rv1016c could enhance the survival of the recombinant *M. smegmatis* within macrophages and promote the resistance to stress factors. Taken together, MTB lipoproteins Rv1016c may be a novel TLR2 ligand and involve mycobacterial virulence.

*Mycobacteria tuberculosis* has been shown to be capable of causing monocytes and alveolar macrophages apoptosis *in vitro*, and alveolar macrophages recovered from MTB patients also showed an increased frequency of apoptosis (Placido et al., 1997; Keane et al., 2000). Recently, considerable interest has emerged in understanding how *M. tuberculosis* infection affects apoptosis of immune cells and whether this contributes to pathogenesis. In the present study, we investigated the potential role of the cell wall-associated protein Rv1016c to induce macrophages apoptosis. Our data showed that soluble Rv1016c alone was able to induce macrophages apoptosis in a both dose- and time-dependent manner, and this effect was specific not due to the His-tag or LPS contamination. TLR2 has been shown to involve apoptosis induced by MTB ligands, and we further examined whether induction of macrophages apoptosis by Rv1016c is TLR2 dependent. The results showed that apoptosis induction by Rv1016c was abrogated in TLR2^−/−^ mouse macrophages or THP-1 cells treated with anti-TLR2 blocking Abs, indicating that Rv1016c may be an apoptosis-inducing factor dependent on TLR2. Taken together, Rv1195 could promote the survival of recombinant *M. smegmatis* and induce apoptosis of host cells, consistent with the effect of MTB19-kDa lipoprotein on macrophages (Lopez et al., 2003), indicating that Rv1016c-induced apoptosis may lead to mycobacterial cell-to-cell spread, and the increased survival of mycobacteria during the infection.

MHC-II molecules continuously expressed in response to infection can load with antigenic peptides in the MHC compartment, and then export to the plasma membrane, where they prime CD4^+^ T cells (Forsyth and Eisenlohr, 2016). Transcriptional control of MHC-II expression may be a molecular basis of immune surveillance, through which APC functions are regulated by *M. tuberculosis* protein during infection (Ramachandra et al., 2009). Actually, MHC-II expression can be regulated by cytokines like IFN-γ or TLRs ligands in macrophages during MTB infection (Stern and Santambrogio, 2016). Some Mycobacterial lipoproteins such as (LpqH and LprG) were found to suppress MHC-II expression via TLR2 in macrophages (Stewart et al., 2005; Shukla et al., 2014). Similarly, in this study, Rv1016c was able to inhibit IFN-γ-induced MHC-II expression and attenuated Ag processing and presentation of macrophages, as reflected by a decrease in IL-2 production (Figure 5). Additionally, Rv1016c-mediated Inhibition of MHC-II expression and Ag processing was reserved in TLR2^−/−^ mouse macrophages or THP-1 cells blocked by anti-TLR2 Abs (Figure 6). Moreover, Rv1016c lipoprotein inhibited IFN-γ-induced MHC-II expression by preventing induction of class II transactivator (CIITA) IV in a TLR2 dependent mechanism (Figure 7). Together, Rv1016c-mediated inhibition of MHC-II expression and Ag processing may lead to the prevention of Ag presentation and the decreased recognition by CD4^+^ T cells. This may provide a mechanism by which intracellular bacilli are able to evade immune surveillance, survive under stress factors and maintain chronic infection.

In this study, we find that cell wall-associated lipoprotein Rv1016c may be a novel TLR2 ligand and may involve mycobacterial virulence by enhancing survival of recombinant *M. smegmatis* within macrophages and increasing resistance to stress factors. Rv1016c may be an apoptosis-inducing factor, and able to promote mycobacterial cell-to-cell spread during infection. Moreover, we determine that Rv1016c lipoprotein may inhibit IFN-γ-induction of MHC-II expression and Ag processing by decreased IFN-γ-induced CIITA IV expression via TLR2 and MAPK signaling. In the context of tuberculosis, we propose that Rv1016c lipoprotein can induce apoptosis, increase the survival of bacilli, and decrease the presentation of MTB Ag to CD4^+^ T cells within human macrophages, allowing intracellular *M. tuberculosis* to evade immune surveillance and promote chronic infection.

## Author contributions

HS, HW, and YX conceived and designed the experiments; HS performed the experiments; HS, YX analyzed the data; HS, ZZ, HW, and YX wrote the manuscript; and HW, YX reviewed the manuscript and supervised the research. All authors read and approved the final manuscript.

### Conflict of interest statement

The authors declare that the research was conducted in the absence of any commercial or financial relationships that could be construed as a potential conflict of interest.

## Abbreviations

Ms: M. smegmatis
TLR: Toll-like receptors
TB: tuberculosis
MTB: *Mycobacterium tuberculosis*
LPS: Lipopolysaccharides
CFU: colony-forming unit
SDS-PAGE: sulfate-polyacrylamide gel electrophoresis
ELISA: enzyme-linked immune sorbent assay
MHC: histocompatibility complex
IFN-γ: Interferon gamma
Pam3: Pam3CSK4.
